# The Fever Nurse

**Published:** 1907-07-06

**Authors:** 


### Nursing Administration.

### THE FEVER NURSE.

The conditions which prevail in the training of nurses in hospitals for infectious diseases are improving every day, and we are face to face with the paradox that the better the training the more difficult the position of these institutions becomes regarded as training schools. In the Metropolitan Asylums Board Hospitals encouragement is given to nurses to supplement their training by the rule that no charge nurse can be appointed who has not had at least one year's training in a general hospital or Poor-law infirmary. A very large number of probationers, or, as they are called in these institutions, second-class assistant nurses, are, however, received who have no previous experience of nursing. These, after serving two years, are passed on into the first class, and it may be presumed that, since without the year's general training already mentioned they are not eligible for further promotion in the fever hospital, they then leave these institutions, having been three years under instruction as nurses, yet without having received training which would give them a passport to employment either in their own special branch of work or any other. What becomes of them?

The Metropolitan Asylums Board has close on 5,000 beds in the fever hospitals in addition to about 2,000 beds for small-pox, and 2,500 beds for convalescing cases. Taking into consideration that the small-pox hospitals are happily only in partial use, and that in the convalescent hospitals the nursing is light, it may be computed that there are at least 1,000 assistant nurses or probationers in training in these institutions. The very size of the buildings renders the nursing administration both difficult and interesting, and the services of able matrons exercised in the best hospital methods are readily secured. The most exact discipline is essential, not only on account of infection, but also on account of the very mixed population gathered within the walls. The training is thus of real value, and the probationer who has served two years in one of these hospitals has been thoroughly well grounded, and is a very different person in the wards to the raw probationer whose position she is, however, expected to take, should she desire to complete her training in a general hospital.

It is growing more and more difficult for nurses to procure without payment a year's training in any general hospital, nor are the Poor-law training schools at all more ready to introduce probationers for a year's course. It may be feared that many probationers drift into private nursing, and form the stock-in-trade of the smaller nursing agencies of whose misdeeds we hear so much. The evil is, as we have said, rendered regrettable from the fact that the training so far as it goes is now generally of a very high order, and attracts a good class of probationer. It is admittedly so essential to the formation of a thoroughly trained nurse that it is usual for nurses aiming at private work to supplement their general training by a course of fever work. Why should not co-operation be possible between the general hospitals and the fever hospitals by means of which fever training could be made part of the three years' course and fever nurses be received in place of the fever pupils to receive general instruction? This is no wild imaginary scheme. It has been tried and found eminently practical and satisfactory by an old-established training school of well-deserved reputation, that of the Royal County Hospital, Winchester. The plan there in force is to keep a second-year probationer for six months at the Borough Hospital, Croydon, to receive instruction in fever nursing, while from Croydon one nurse is received who gets a year's training in general nursing at Winchester. It will be seen that for every nurse who is trained at Winchester, two probationers can receive six months' fever training at Croydon, the fever hospital paying salaries to these pupil nurses. The plan is found to work excellently in all respects.

The probationer, after her change of work, settles down again contentedly among her old companions, aware that she is well on the road to become a useful member of the private staff, while the advantage to the fever nurse needs no enforcing. Care is always taken to select promising probationers for these exchanges, and the prospect of being chosen for the position of honour works as an incentive to effort. The plan has, indeed, proved so advantageous that it has been expanded so that pupils are exchanged between the National Hospital, Queen Square, and the Winchester Hospital, for six months at a time, while at the Southampton Eye Infirmary a pupil from Winchester is often received also for three months' special training.

There can be no doubt that this system entails a good deal of thought and care on the part of the matron if it is to be a success. It also demands the power of appreciating the value of other people's work and methods. Those who never look into the households of their fellow-workers without disparaging their labours, would deem it deplorable that any probationer should pass away from under their direct influence during the time of training. But mutual comprehension and mutual trust should do much to destroy this feeling of suspicion with regard to other hospitals, and, carried out under proper precautions, the system of exchanges can but result in widening the minds of pupils and superintendents alike. It tends to promote just that sense of cordial co-operation which is sadly lacking, as a rule, among the heads of institutions, and to introduce a welcome variety into the training of the smaller general hospitals. As a solution of the problem what to do with the half-trained fever nurse, its value is incalculable. The only way in which the weak links in the present training system can be strengthened is by interdependence among hospitals presenting different varieties of nursing experience, and every attempt in this direction is to be welcomed.

